# The impact of the war in Ukraine on the well-being of German medical students: a mixed-methods study

**DOI:** 10.1038/s41598-025-86861-8

**Published:** 2025-08-05

**Authors:** Marie Stelter, Michaela Zupanic, Angelika Taetz-Harrer, Julia Nitsche, Arndt Büssing, Jan P. Ehlers

**Affiliations:** 1 School of Didactics and Educational Research in Health Care, Witten/Herdecke University, Alfred-Herrhausen-Straße 50, 58455 Witten, Germany; 2 School of Personal and Interpersonal Skills training in Health Care, Witten/Herdecke University, Alfred-Herrhausen-Straße 50, 58455 Witten, Germany; 3 School of Integrative Medicine, Witten/Herdecke University, Alfred-Herrhausen-Straße 50, 58455 Witten, Germany

**Keywords:** Stress perception, Work engagement, Medical students, War events, Resilience, Social support, Psychology, Health care, Risk factors

## Abstract

The ongoing aggression war by Russia in Ukraine underscores the need to explore the impact of geopolitical crises on students’ well-being within the system university. Previous studies have raised concern regarding rising mental health issues among students, exacerbated by war-related stressors. The effects extend to non-war-involved countries on several levels, with heightened anxiety and fear reported for students. In this study, the affectedness of well-being, perceived stress and work engagement among German medicine students is surveyed in a cross-sectional multi-methods study before and after the initial war occurrences in Ukraine were reported. Results show lessened well-being and increase perceived stress, but non significantly affected work engagement. The students report several demands and resources to cope with stressors and maintain ongoing study motivation and capability. By understanding the personal repercussions of systemic crises, this study seeks to identify longitudinal support needs for medical students navigating uncertain times.

## Introduction

It has been known for a long time and has been very well studied that the study of medicine places great demands on students. These requirements are particularly evident in relation to the intellectual capacity and the discipline of the students, which is why very different selection procedures for medical studies have been established worldwide. These are intended to ensure the success of studies by enabling students to acquire the competencies (knowledge, skills, attitude) necessary for the medical profession. In addition, medical students are expected to bring certain personality and resilience factors to face and successfully complete demanding study periods and clinical work requirements, such as conscientiousness or self-efficacy^1,2^.

With respect to rising environmental, man-made crises in addition to those happening within the healthcare system, as global pandemics, wars, and military conflicts as well as nuclear threats and occurrences due to climate change constitute, this person-centred perspective might turn into a cynical one^3,4^. Besides the biological or trained potential, a student provides, social and environmental factors may seal whether the existing potential of a person can be realized or not^5,6^. According to Ecological Systems theory, a person’s behavior and wellbeing is not only determined by the individual, but on five levels in interaction over the lifetime^7^. On the micro-level, a student might be influenced by occurrences in the close family and university setting. The intersecting meso-level includes interactions with family and/or between university professors, counsellors, and other contributors. Social media interactions may be classified here as well^6^. Global environmental stressors can be reflected on the exo-level, which represents less direct experiences for the student.

These experiences might be linked to increased stress experiences as well as decreased psychological resources to study in students^8,9^. The COVID-19 pandemic, as an example for a stressor associated with the exo-level, decreased the psychological wellbeing of students, and affected the stress perception at the Witten/Herdecke University (UW/H) previously^10^. The explanation for these experiences cannot be given by the dissatisfaction with their medicine studies or possible content with distance teaching formats during the pandemics alone, as the micro-level or meso-level would indicate. Therefore, other system levels appear to influence the students’ satisfaction further. Other socio-cultural studies with elite student’s state that students are affected by all levels described in relation to their personal identities, aims and motivation^9^. Hence, systemic crises may have an additional influence on the psychological wellbeing of medicine students as well as the stress perception in different phases of studies, indicating a need for special coping strategies and support for the student^11^.

Current global wartime events may constitute such a form of systemic crisis^12^. Depending on the level of affectedness (here: by the war), related events can have a substantial influence on the mental and physical health of people as well as the capability to face study or work duties^13–15^]. In response to the aggression war started by Russia in Ukraine, which began on February the 24th 2022, many people’s mental health also outside Ukraine were severely affected^15,16^. Especially adolescents and young adults constitute high-risk groups to develop mental disorders according to the WHO, which is why there is a general focus of interest to analyze the consequences of crisis in the health of young people^16,17^. There are reports of increased risk of mental health consequences for young people affected by war time events such as PTSD (post-traumatic stress disorder), depression and anxiety^18,19^. Riad and colleagues examined the effects of the Ukrainian war on Czech republican students and found these effects evidenced by high rates of “feeling concerned” correlating with increased rates in anxiety and depression measures over different age groups, genders and study fields^16^. Other studies including Polish people and refugees from Ukraine of young people and others show high rates of war anxiety as well as persistent thinking of war, both measures correlating with PTSD symptoms and depressive symptoms^20,21^. The health of Ukrainian students is linked to increased measures of depression, loneliness, nervousness as well as substance abuse in the consequences of war times^20,21^. The individual capability and motivation to study may be also affected in the light of war perils^24,25^. It could further be shown that not only students are affected by the war situation in Ukraine, but also the exo-system University in war-related countries itself by facing scarcity of educational resources, propaganda-induced teaching and subsequently twisted facts as well as the individual fear of losing national identity^24,26^.

Besides the influence of direct war occurrences on people living in regional proximity, there might be effects on people in non-directly war-involved countries as well. This may be constituted by reinforced insecurity through economic impacts, sensational-based media coverage of the war situation or mass exodus^13,27–29^. There are reports of increased levels of helplessness in Italian samples as well as other negative emotions including anxiety, anger, or disgust in consequence to war occurrences in Ukraine, leading to possible consequences by prolonged exposure for public health^30,31^. In Germany, as a country with a history of war, these symptoms can be found in financial concerns as well as the fear of being involved in war acts^29,32^. In the first quoted study, the fear of a conventional and nuclear war could be associated with frequent social media use, indicating a reinforcing factor for the fear of war by news and possible mediating risk factor for mental health in non-directly war-involved countries.

The scope of these influences of systemic crisis on individual health as well as the success and study satisfaction in times of uncertainty might depend on the given personal prerequisites of a student as well as structural study demands and resources^33,34^. The Job Demands-Resources Model (JD-R Model) describes the relationship between demands referring to personal psychological costs, as well as resources that can be beneficial for achieving individual goals, reducing demands, or promoting personal growth^33^. The strain of the work as well as personal motivation are affected by these processes and may influence the outcome of the work as well as stress-related health consequences. More specifically, the interaction of resources and demands can result in buffering the effects of demands on job strain, increasing the potential to reduce stress-related personal problems like Burnout^35^. Possible job demands can be seen in work overload and emotional challenges, which might result in the scarcity of mental and physical resources of the worker and furthermore energy exhaustion. For medical students, similar demands may result in psychic and psychosomatic consequences in different study phases^11^. Among students of the UW/H, an increase in the request of psychological support, decrease of study contentment as well as the onset of psychosomatic symptoms especially with the beginning of the clinical phase reflect possible job or study demands in times of increased volatility of a student’s career^11^. Possible stress-buffering resources are found mostly in social support, the understandability of tasks and the perceived controllability or autonomy, indicating a possible need for these qualities in the mentioned study phases^33^.

It is not yet known but reasonable to assume that individual study demands as well as stress-buffering resources are negatively affected by uncontrollable events on the exo-level like the aggression war started by Russia in Ukraine, implicating the utility of a modified job-demand-model to examine both personal and external influences on individual wellbeing and study resources of medicine students at the UW/H^10^. As called for by the authors of Riad and colleagues^16^ the reasons for feeling concerned in a country not directly involved in the war and the resulting accelerated systemic demands on students, especially in stress-prone phases of their studies, are surveyed in order to identify the need for support in times of volatility in the study of medicine at the UW/H. Consequences of these demands are reflected in the personal well-being and perceived stress as well as the effects on study motivation, represented by the work engagement of the medicine students. The strengths of this study reflect the so far insuffiently examined systemic effects on wellbeing and job efficiency in students from a non-directly affected, but global perspective. The questions of interest for the following study are: What impact did the war in the Ukraine have on medical students at UW/H? What resources do they report to cope with the new stress?

## Method

### Sample

Since the winter semester 2018/19, medical students at UW/H have been regularly surveyed about their well-being, stress perception, and work engagement using an online questionnaire^10^. The respective ReBel study was approved by the ethical commission of Witten/Herdecke University (#132/2017). The study was performed in accordance with relevant guidelines and regulations regarding the qualitative and quantitative questioning of human participants^74,75^. In this study, we consider two groups of medical students who completed the questionnaire at the beginning of the war in the Ukraine in March 2022 and at the end of the summer semester 2022 during the respective survey period of three weeks. Two groups of medical students at UW/H have completed the ReBel questionnaire in the respective survey period of three weeks.

### Measures

In accordance with the ethics committee and the data protection officer, all important information on data protection, confidentiality and anonymisation was listed on the start page of the questionnaire. Participants had to actively agree to this via a radio button in order to take part in the survey. If the data protection provisions were rejected, the respondent was redirected to a neutral page (university homepage). The anonymous online questionnaire used the following standardised measures in addition to the required basic sociodemographic and semester data: The 10-item Perceived Stress Scale (PSS)^36^ was used to collect subjective stress perceptions. All items refer to emotions and thoughts in the past month, answered on a four-point Likert scale ranging from 1 (never) to 4 (very often). The internal consistency of the scale was good in medical students from Germany (Cronbach’s alpha = 0.89)^10^. Subjective well-being in the past two weeks was assessed using a scale ranging from 0 (never) to 5 (all the time) as the WHO-Five Well-being Index (WHO-5), where scores above 13 may indicate depressed mood^37^. The internal consistency of the WHO-5 in the German version is described as very good (Cronbach’s alpha = 0.92)^38^. Work engagement was measured with the Utrecht Work Engagement Scale in the shortened version with nine items (UWES-9)^39^. Items are answered on a seven-point Likert scale ranging from 0 (never) to 6 (always/every day). The internal consistency of the three subscales, vigor, engagement, and absorption, ranges from 0.85 to 0.92. At the end of the online questionnaire, an open-ended question asked about the current state of mind of the medical students: “Here you have the opportunity to write down in your own words your feelings about particularly stressful situations. What do you do to deal with them better? What are your strategies and initiatives for dealing with particularly stressful situations?” The responses were analyzed with a summary qualitative content analysis according to Mayring^40^ and are reported with the number of mentions in the categories. The quality criterion of intersubjectivity is fulfilled by consensual coding with subsequent discussion among the authors^41^.

### Statistical analyses

Descriptive statistics are reported as frequencies for categorical variables (number and percent) and as mean ± standard deviation (M ± SD) for numerical variables. Comparisons between the two groups were made using Pearson’s Chi2 test of independence for categorical variables and nonparametric Mann-Whitney U tests for numeric variables using SPSS version 29. Results are considered significant with an α-value of *p* < 0.050.

## Results

In March 2022 out of 179 responding students, 156 (87.1%) can be considered with complete data as group 1, in July 2022 out of 598 responding students 524 (87.6%) as group 2 for comparison. The students of group 1 and 2 don’t differ in proportion of gender, mean age, mean semester or section of studies (Table 1). There are more female students than male students in both groups with a trend for more women in the preclinical semesters. Male students are less represented in group 1 in the preclinical section (44.4%), and more represented in group 2 in the clinical section (55.6%) than women (52.1% resp. 47.9%), without this difference reaching statistical significance.

**Table 1:** Description of medical students of group 1 and 2.

	Students group 1 (March 2022)	Students group 2 (July 2022)	*p*-value
Number	156	524	
Gender (#, %)			
Female	101 (64.7%)	329 (62.8%)	n.s.
Male	52 (33.3%)	191 (36.5%)	
Divers	1 (0.6%)	3 (0.6%)	
Missing	2	1	
Age (years) (m ± sd)	24.8 ± 2.8	24.6 ± 2.9	n.s.
Semester (m ± sd)	5.2 ± 2.5	5.0 ± 3.2	n.s.
Phase of study (#, %)			
Preclinical (1–4)	64 (41.0%)	270 (51.5%)	n.s.
Clinical (5–13)	92 (59.0%)	254 (48.5%)	

Significant differences between groups 1 and 2 in subjective well-being (WHO-5), perceived stress (PSS), and work engagement (UWES-9) are evident (Table 2). Medical students reported significantly less favorable scores in the survey immediately after the start of the Ukraine war in March 2022 (group 1), with poorer well-being, higher perceived stress, and a tendency toward lower work engagement. More than half of them had a wellbeing score of < 13 (WHO-5), which could indicate depressive mood states. In the survey four months later in July 2022, medical students (group 2) had a significantly better well-being, lower perceived stress, and tending to have improved work engagement. Significantly fewer than in group 1, namely about one third, had a WHO-5 score below 13 points.

**Table 2:** Wellbeing (WHO-5), perceived stress (PSS) and work engagement (UWES-9) of medical students of group 1 and 2.

	Students group 1 (March 2022)	Students group 2 (July 2022)	Significance *p*
Wellbeing (WHO-5) (m ± sd)	11.6 ± 5.5	15.0 ± 5.0	< 0.001
WHO-5 scores < 13 (#, %)	91 (58.3%)	186 (35.5%)	< 0.001
Perceived Stress (PSS) (m ± sd)	29.8 ± 7.1	25.2 ± 6.5	< 0.001
UWES-9 Vigor (m ± sd)	3.4 ± 1.1	3.6 ± 1.1	n.s.
UWES-9 Dedication (m ± sd)	3.5 ± 1.1	3.6 ± 1.1	n.s.
UWES-9 Absorbtion (m ± sd)	3.6 ± 1.3	3.8 ± 1.2	n.s.
Work Engagement (UWES-9) (m ± sd)	3.5 ± 1.1	3.7 ± 1.0	n.s.

**Fig. 1:**
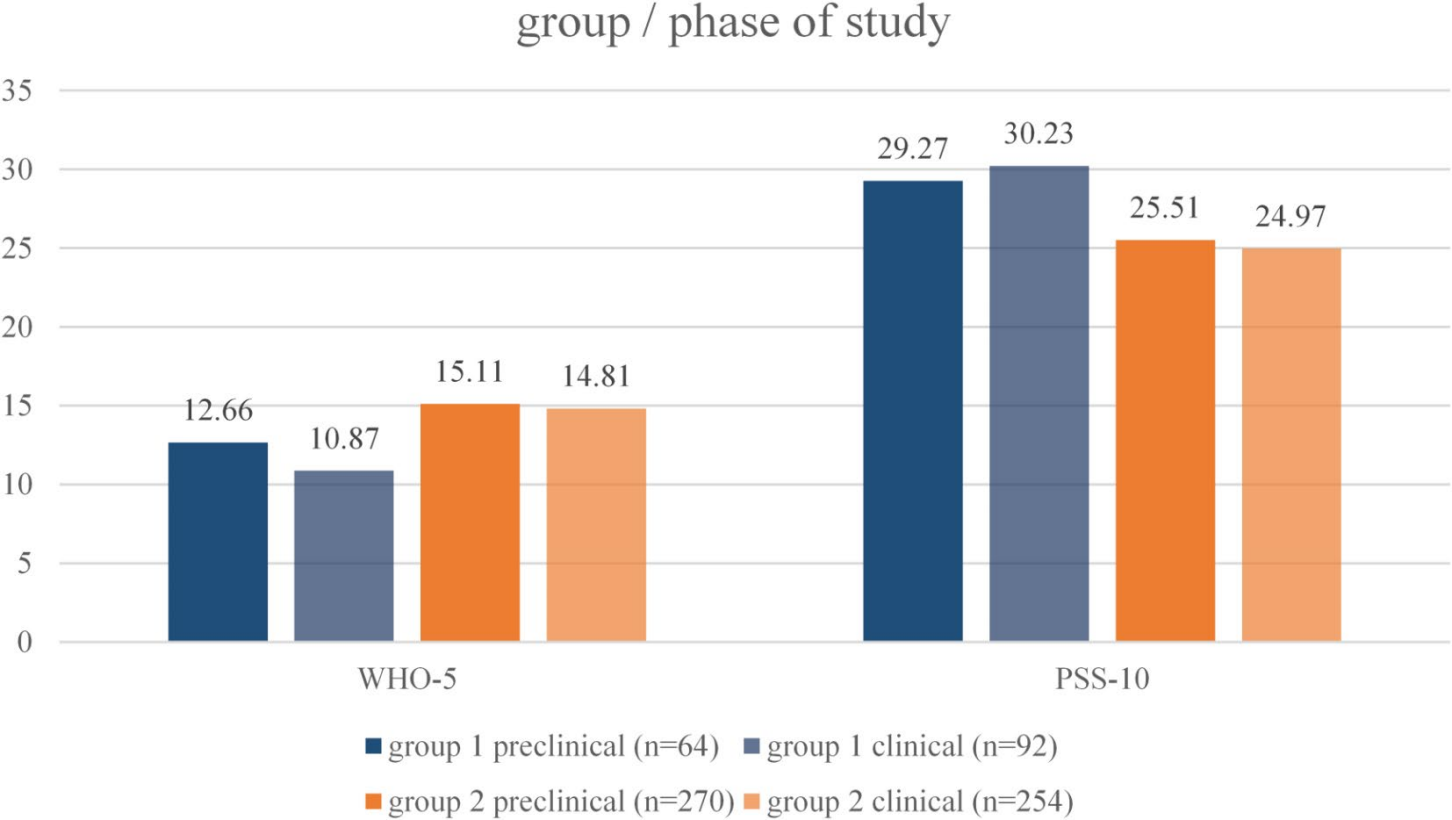
Wellbeing (WHO-5) and perceived stress (PSS-10) in Students Group 1 (*N* = 156) and 2 (*N* = 256) for study phase.

The results of both groups for WHO-5 and PSS are shown Fig. 1, differentiated by study phase. There is a slight decrease in subjective well-being and an increase in experienced stress with entry into the clinical phase in group 1 from March 2022.

In group 1 of March 2022, a total of 39 medical students (25.0%) answered the open-ended question. Nine categories could be extracted with content-analysis, four of which could be assigned to each of the two domains according to the Job Demands-Resources-Model^31^ and one category Other. A total of 82 codings were made. Demands were Strong emotions such as fear, horror, and anger (#19 mentions), followed by helplessness/powerlessness (#8), learning disabilities (#6), and perceived lack of support (#4). Strong emotions and desire for support from the UW/H are evident in the following quote:

*“It’s unbelievable that there is a war going on in Europe. It is devastating and makes one feel very sad. There is well-founded concern that the war will spread to NATO territory and that Germany will also be directly affected. Is there an emergency plan on the part of the university?” (Student 14)*

Distraction and conversations with friends (#14 mentions) were named as resources, followed by seeking information in the news about the war (#13) and active help for refugees (#13) as well as media abstinence (#11). Dealing with stresses and a corresponding counter-regulation is evident in this quote:

*“I try to reduce the feeling of powerlessness by taking action. For example, by collecting donations, offering shelter, and volunteering to help refugees arrive. This helps me a bit with dealing, but the dire and uncertain situation weighs heavily on me. Some days I have a high urge to read/watch news. On other days, I consciously take a break to “give myself a break.” I bought extra flowers and walk in the sun and consciously talk about other things than the war and suffering. (…) (Student 21a)*

The category Other (#5 mentions) includes political statements that could not otherwise be assigned, such as the following quote:

*“In such an unbelievably bad situation and possibly with incalculable outcomes and escalation levels, one should act prudently and wisely, and for me that includes good communication with the population, because in the end, democratic power lies with us and in the end, it is we who must bear the consequences.” (Student 17)*

In group 2 of July 2022, only 13.5% of the medical students (*N* = 71) answered the open-ended question, considerably less than in group 1 with a response of one-quarter of the students. With the content analysis nine categories could be extracted as well, six in the domain Demands, two Resources and one category Other. A total of 54 codings were made. The demands mentioned were exam stress (#5 mentions), followed by problems in the clinic/with patients (#4), world events with pandemic and Ukraine war (#3), perceived lack of support (#2), and need for rest (#2). The Strong Emotions prominent in Group 1 were mentioned by only one student (#1) and attributed to personal reasons. Problems in the clinic are evident in the following quote:

*“It’s not the academic situations, it’s the hospital situations. The structures are still (also very much abroad) clearly hierarchical and unfriendly to people. This is stressful; I have learned to distance myself.” (Student 19)*

The most frequent responses concerned resources for dealing with stress in the form of distraction and conversations with friends/colleagues (#21 mentions), followed by the response that there is no current stress (#10). The latter is vividly illustrated in the following quote:

*“I find the study (preclinical) was so far in no way that it has burdened me badly. I always had a lot of free time, the life stress is of course not completely gone with the amount, but the study enriches me much more and does me very well above all also if I’m not doing well privately.” (Student 31)*

The category Other (#6 mentions) includes socio-political statements that could not otherwise be assigned, such as the following quote:

“*Especially the punk “service by the book” concerns me (…) A lot of what we learn in theory in college is only done in practice in very stripped-down versions. It helps me to talk to my instructors and find out whether it is the normal situation and how they deal with it in the long run, always doing only what is necessary, not fulfilling everything and making mistakes. Especially working in a team then gives me a better feeling.” (Student 21b)*

**Fig. 2:**
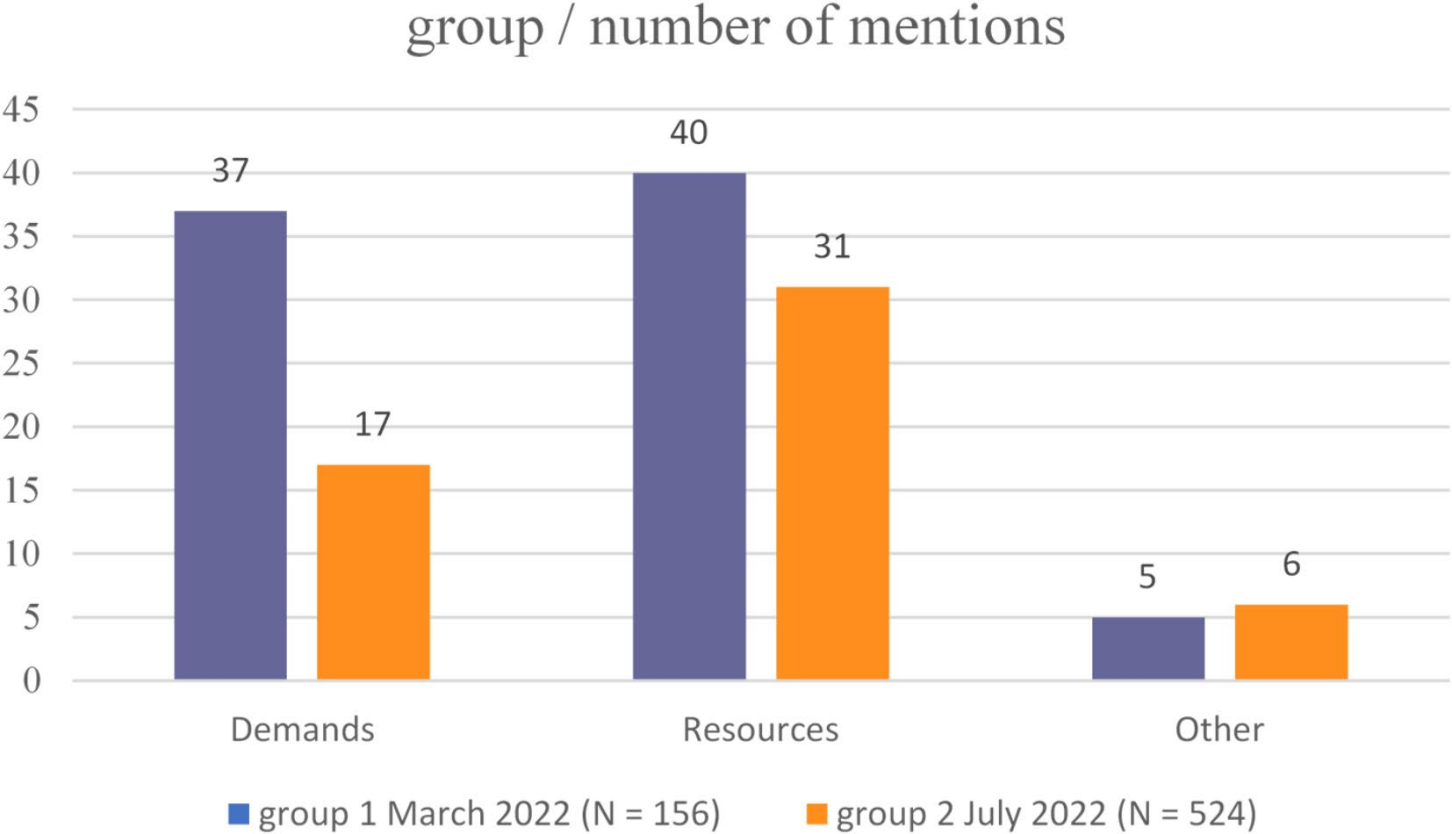
Number of mentions in Group 1 (*N* = 156) and 2 (*N* = 256) for domains of the JD-R Model.

When comparing the two groups based on the frequency of mentions in the two domains according to the JD-R Model, differences become apparent and are documented in Fig. 2. While in group 1 both domains are represented with a comparable number of mentions, group 2 mentions fewer demands.

## Discussion

The findings indicate that medical students of the UW/H are affected by the aggression war act of Russia on Ukraine 2022. The findings are comprehensible with respect to the decreased wellbeing, stress perception and study capabilities as previously shown by the medicine student cohort during the COVID-19 pandemic with missing link to the contentment with the studies or university support alone^10^. However, students’ stress perception decreased during the COVID-19 semester, whereas it increased during the time after the report of the war in Ukraine in this study. This is also shown in other cohorts of students of different study fields and may be a key argument for the structural relevance and importance of the consideration of systemic volatility in stress perception, which might decrease in times of cultural, economic and general contact restrictions during the pandemic in contrast to “just” empathy-based concernment and anticipating fear, that the war will also affect Germany in the future after the war act on Ukraine^27,29,42,43^. Interesting to note is that the students of the UW/H seem to regulate their emotions (here: after a time span of 4 months) in a comparable way, showing favorable scores in wellbeing over time^10,42,43^. This will be discussed for further research and student support with a view to the reinforcement of media reports and crisis communication, individual affectedness of the events on the exo-level as well as individual study characteristics on the micro- and meso-level^7,10,13^.

On the individual level, people tend to process critical information along the dimensions of attending, reacting, explaining to successfully adapt and integrate the information in used schemes^13^. The attending phase is influenced by the novelty of information and the self-relevance, which in this German sample may be assessed as highly relevant due to the lack of war acts in Europe since 1945^44^. When self-relevant events are highly surprising and not (yet) fully explainable or controllable, they tend to be highly accessible in memory, leading to possible anxious or intrusive thoughts^45^. This may be reflected by answers to openly asked questions regarding current stressors (e.g. “It’s unbelievable that there is a war going on in Europe. It is devastating and makes one feel very sad (.)”, Student 14) in this study and the negatively affected emotional state in March 2022 right after the first war events happened (#19).

The interaction of the external happenings to own competing goals and concerns influence the self-relevance of the (here: war-related) information, which may be embedded in the demanding study of medicine for the students of the UW/H in the micro- and meso-level^6^. Therefore, the adaptation results of restored wellbeing, stress perception and work engagement after 4 months in student group 2 may be explained by moderating influences of study demands and goals, which constitute more demanding self-relevant concerns as time passes by^13,14^. The insecurity-inducing news and circumstances of the war might get more explainable and usual over time, yet are not directly perceivable, whereas the study goals stay relevant and demanding^14^. This could explain the restored levels of well-being in the second study group with the course of the war in Ukraine. Another regulation aspect may be the social amplification and risk framework, indicating that risk perception is influenced by media coverage especially when the happenings are not perceivable by oneself but transferred via media processing to a non-directly affected person^14^. Relevant here is the social communication, which constitutes a subjective interpretation of the event and may bias the message as well as the risk perception by exaggerating emotional aspects in favor of attention by keeping events and the medium current and popular^46,47^. Individual efforts to fend off negative affectedness from media (media abstinence, #11) in this study suggest that the students are aware of the negative influence of news and might actively try to regulate their news consumption and the inducing of negative feelings, resulting in possible restored wellbeing through avoided negative news. The scope of individual consumption of media, the format and (negative) tone of the (journalistic) provider as well as social media actors in general remain unknown influencing factors for the felt affectedness of students in non-directly war-related countries^15,29,48^.

The described risk framework may be both applicable in comparison to previous findings and related to self-relevance during the COVID-19 pandemic, from which the students may be constantly or more affected over the time, as the infection rates and the scope rose and consequences were perceived more directly^10^. This may be a key point and argument for understanding the differences in stress perception during different types of crises along individual gradients of self-relevance and ongoing event reports as well as health consequences for students^14,49^.

According to these theories, there might be another explanation for the adaptation of wellbeing, risk perception and work engagement with ongoing war events and other bad news. Due to the theory of motivation protection and trauma-like stress events, people only have limited resources of worry and will eventually numb and avoid negative news with accumulating scope and without increasing resources or possibilities to influence the events by themselves, resulting in emotionally defending the stressor events as an emotion regulation strategy^50,51^. In this study, students of group 1 showed patterns of fear, horror, and anger (#19), along with helplessness/powerlessness (#8), indicating a high amount of negative emotional demands and limited felt self-efficacy to change the situation. This short-term regulation of well-being may demonstrate a defensive coping reaction and it might had happened in Group 2 due to limited engagement possibilities to influence the war events. Matching this on the micro- and meso-level, students of the clinical phase report a significant higher stress perception and lessened wellbeing than pre-clinical students, which is a study phase associated with increased insecurities due to patient contact and hopelessness due to increased volatility of social expectations, raising personal requirements and hierarchical work processes in the clinical work environment, especially for women^52–54^. The example quote “The structures are still (also very much abroad) clearly hierarchical and unfriendly to people. This is stressful; I have learned to distance myself.” (Student 19, clinical phase) may reflect this way of emotional processing. Hopelessness and worry, according to other research, constitute a vulnerability for mental health consequences such as depression after stressful life events [cf. “learned helplessness”,^30,50,55,56^]. In this study, students appear to regulate their stress-related symptoms on subclinical depression scope to war events in Ukraine after wellbeing restrictions on subclinical depression scope but long-term effects of accumulated stressors and emotional numbing, especially in challenging study phases or combined with personal crises remain unclear.

In this study, the non-significant changes in work engagement even with the enduring war events within four months might reflect an interesting resilience factor for this felt hopelessness. The course of stable work engagement is comparable to that during the COVID-19 pandemic^10^. Work engagement might constitute a time-stable factor for the studies also for German private medicine students, as previously connoted as a stable and support-worthy resource for the success of job-related tasks and regulation of demands among workers^35,57^. However, besides work-related outcomes, work engagement may have the power to increase self-efficacy, which is demonstrated as a mediating resource for the influence of stress during crises like the COVID-19 pandemic, lessening feelings of hopelessness in the face of multilevel crises^58^.

Students of medicine at the UW/H already report several other strategies or resources to deal with hopelessness and other demands. These might be classified as actively problem-oriented or beneficial for the experience of self-efficacy (seeking information in the news about the war, active help for refugees) as well as passively coping-oriented or beneficial to emotion regulation (distraction and conversations with friends, media abstinence), suggesting the transferability of the findings in the light of reported theories and literature. In other studies, individual regulation from social support as well as participating and thinking solutions rather than problem-oriented, are reported as main resources for stressful events, too^59,60^. Statements like “I try to reduce the feeling of powerlessness by taking action. (.) This helps me a bit with dealing, but the dire and uncertain situation weighs heavily on me (Student 21a) indicates at least partly successful engagement in active stress regulation behavior of students at the UW/H. In the context of the JD-R-Model, these contribution of resources to the capability to study of the medicine students as well as the unaffected work engagement may be explained, indicating a regulating effect of the described resources on individual motivation, and felt strain of the given study demands^33^. Other positive coping strategies reflected by the literature may be a positive reframing of events, humor, acceptance, or religious behavior^61,62^. Techniques from the acceptance and commitment therapy, mindful-based therapy or cognitive behavioral therapy appear to additionally decrease symptoms of distress, anxiety, or depression in times of crisis^63^. Mindful-based techniques as a coping mechanism may already be applied by the students, reflected by statements like (.) I bought extra flowers and walked in the sun and consciously talked about other things than the war and suffering. (.) (Student 21a). Behavioral strategies may be shown by situate coping principles and active exposure behavior as statements like (…) I try to reduce the feeling of powerlessness by taking action. For example, by collecting donations, offering shelter, and volunteering to help refugees arrive (.) (Student 21a) indicate^64^. These overall findings demonstrate a stress regulation capability of medical students in times of crisis but represent support areas for the university in order to facilitate the individual engagement in medical studies with increased external hopelessness as multilevel demands may cause^33,65,66^.

Research indicates that there might be other possible interfering factors on the systemic or individual levels influencing the wellbeing and stress perception of students, possibly limiting the given explanations and true associations to the war events^67^. On the exo-level of the universal studies, the given results indicate differences of e.g. stress perception in clinical vs. preclinical phases of medical studies Fig. ure 1). It is unclear how different factors of the clinical environment and factors of different study phases might covariate with the individual or well-being of the study cohort or stress perception and further research could try to depict influencing factors on multi levels in order to separate personal or study wise influences from those of the war events^68,69^. On the individual level, previous research suggests covariability of wellbeing and stress perception of students in times of crises in political ideology, gender, phases of study, social media use, the individual capability of emotion regulation and endurance of insecurity as well as other resilience factors^10,70^. Due to a personal selection process for medicine studies at the UW/H, personality factors may additionally influence individual crisis processing, possibly limiting the transferability of the findings to other samples^71,72^. The mentioned factors may moderate the original relation of the war events, study engagement and reaction measurements, limiting generalizing statements about the effects of the war on UW/H students and their studies. To improve the validity of the findings, a suitable methodological consideration of these influences may be given by including reported possible confounding variables as media consumption, social media or media agenda in stepwise regression analyses or multilevel models to distinct inner and outer influences of stressors over study programmes on the individual^69^. In reaction to these findings, the university actively strives to reflect on and adapt the support and counseliing structures—such as academic advising, mentoring, initiative-based activities, and informational events—based on the results on the ongoing longitudinal survey, ensuring that these services align with students’ needs and provide optimal support, even and especially during times of crisis.

## Conclusion

Overall, medicine students appear to be emotionally affected by systemic, environmental crises, but also demonstrate resilience and stable engagement resources for study success. Students request social support from the university in order to deal with external and study wise (health) stressors. Further research should survey on how to support self-efficacy of students in times of felt hopelessness and increased volatility on several levels around the student in order to prevent health problems and ensure innovative and enduring health practitioner behavior in future demanding work phases^65^. This study reflects stable resources of work engagement of medicine students, indicating a predictor for ongoing study dedication as well as solid commitment to the university and the studies at the UW/H^10^. Examining factors such as self-relevance and influences of crisis communication, social media use and media-related exposure to that variable may contribute to a clearer understanding of the complex influences of systemic crisis on individual students’ health and career in the future^11,48^. Moreover, further research could include the effects of the social system of the university and the teaching itself in order to model complex interactions of social and cultural events on the quality of universal teaching and embedding of human crises in medical education. Evidently, the demands and resources regarding the war events reported by the students reflect the need of social support culture on the level of the university, which is already structured and strived to continue by providing rooms for debriefing, emotional exchange and consolation among students and employees in the face of crises and demanding study phases at the UW/H. When applied to broader university frameworks fostering a comprehensive culture of social support should be priotized. Practical measures include the establishment of structured peer-support and resilience programs, such as mentoring systems, where senior students guide peers during crises. Additionally, offering facilitated discussion groups and regular debriefing sessions led by trained professionals could help mitigate stress and enhance emotional resilience. The results also underline the importance of embedding long-term strategies, such as integrating mental health resources and resilience-building workshops into the university’s standard support offerings [as examined by Abulfaraj and colleagues,^73^]. By actively reflecting on and addressing the dynamic needs of students and employees, universities can create a sustainable framework that enhances well-being during crises and throughout demanding academic phases.

## Data Availability

The datasets analysed during the current study available from the corresponding author on reasonable request.
